# Differences in Bone Metabolism between Children with Prader–Willi Syndrome during Growth Hormone Treatment and Healthy Subjects: A Pilot Study

**DOI:** 10.3390/ijms25179159

**Published:** 2024-08-23

**Authors:** Joanna Gajewska, Magdalena Chełchowska, Katarzyna Szamotulska, Witold Klemarczyk, Małgorzata Strucińska, Jadwiga Ambroszkiewicz

**Affiliations:** 1 Department of Screening Tests and Metabolic Diagnostics, Institute of Mother and Child, Kasprzaka 17a, 01-211 Warsaw, Poland; magdalena.chelchowska@imid.med.pl (M.C.); jadwiga.ambroszkiewicz@imid.med.pl (J.A.); 2 Department of Epidemiology and Biostatistics, Institute of Mother and Child, Kasprzaka 17a, 01-211 Warsaw, Poland; katarzyna.szamotulska@imid.med.pl; 3 Department of Nutrition, Institute of Mother and Child, Kasprzaka 17a, 01-211 Warsaw, Poland; witold.klemarczyk@imid.med.pl (W.K.); malgorzata.strucinska@imid.med.pl (M.S.)

**Keywords:** Prader–Willi syndrome, Gla-osteocalcin, Glu-osteocalcin, periostin, insulin-like growth factor-I, total-IGF-binding protein-3

## Abstract

Despite therapy with growth hormone (GH) in children with Prader–Willi syndrome (PWS), low bone mineral density and various orthopedic deformities have been observed often. Therefore, this study aimed to analyze bone markers, with an emphasis on vitamin K-dependent proteins (VKDPs), in normal-weight children with PWS undergoing GH therapy and a low-energy dietary intervention. Twenty-four children with PWS and 30 healthy children of the same age were included. Serum concentrations of bone alkaline phosphatase (BALP), osteocalcin (OC), carboxylated-OC (Gla-OC), undercarboxylated-OC (Glu-OC), periostin, osteopontin, osteoprotegerin (OPG), sclerostin, C-terminal telopeptide of type I collagen (CTX-I), and insulin-like growth factor-I (IGF-I) were determined using immunoenzymatic methods. OC levels and the OC/CTX-I ratios were lower in children with PWS than in healthy children (*p* = 0.011, *p* = 0.006, respectively). Glu-OC concentrations were lower (*p* = 0.002), but Gla-OC and periostin concentrations were higher in patients with PWS compared with the controls (*p* = 0.005, *p* < 0.001, respectively). The relationships between IGF-I and OC (*p* = 0.013), Gla-OC (*p* = 0.042), and the OC/CTX-I ratio (*p* = 0.017) were significant after adjusting for age in children with PWS. Bone turnover disorders in children with PWS may result from impaired bone formation due to the lower concentrations of OC and the OC/CTX-I ratio. The altered profile of OC forms with elevated periostin concentrations may indicate more intensive carboxylation processes of VKDPs in these patients. The detailed relationships between the GH/IGF-I axis and bone metabolism markers, particularly VKDPs, in children with PWS requires further research.

## 1. Introduction

Prader–Willi syndrome (PWS) is a rare congenital neurodevelopmental disorder characterized by hyperphagia, growth hormone (GH) deficiency, short stature, low bone mineral density, and various orthopedic deformities, including scoliosis, kyphosis, hip dysplasia, and flat feet. Both adolescents and adults with PWS show a high prevalence of osteoporosis and fractures [1]. In children, PWS is associated with disruptions in the growth hormone axis and bone turnover, potentially impacting bone mass in later life [2]. However, the exact mechanisms underlying bone disturbances in PWS remain unclear. Notably, while some studies suggest that GH treatment improves bone mineral density (BMD) in PWS patients [3], others report a gradual decline in the BMD standard deviation score (BMD-SDS) despite long-term GH therapy [4,5].

Osteocalcin (OC) and periostin are vitamin K-dependent proteins (VKDPs) critical to both bone and vascular tissues. Their biological functions rely on a vitamin K-dependent post-translational modification, where specific glutamic acid (Glu) residues are converted into gamma-carboxyglutamic acid (Gla) residues [6]. Periostin—an extracellular matrix protein primarily expressed by osteoblasts and osteocytes—enhances osteoblast differentiation and proliferation, inhibits β-catenin degradation via sclerostin Wnt signaling, and promotes collagen cross-linking through lysyl oxidase activation [7]. Elevated serum periostin levels have been linked to higher fracture risk in postmenopausal women [8]. In bone formation, osteocalcin plays a pivotal role in organizing the bone extracellular matrix and exists in two forms: carboxylated osteocalcin (Gla-OC) and undercarboxylated osteocalcin (Glu-OC) [9]. Osteocalcin’s Gla residues confer a high affinity for Ca^2+^ and hydroxyapatite in the mineralized bone matrix. The undercarboxylated form, which is biologically active, is released from osteoblast secretion and partly from osteoclastic bone resorption. The ratio of circulating carboxylated to undercarboxylated osteocalcin reflects vitamin K status [10]. Additionally, osteopontin (OPN)—another key non-collagenous protein in the bone extracellular matrix—plays multiple roles in bone metabolism by regulating bone size and density [11]. OPN promotes osteoclast adhesion to the bone matrix, thus regulating bone resorption. Its levels are significantly higher in children compared with adults, and increased in obese children relative to their lean counterparts [12,13]. Osteoprotegerin (OPG) and sclerostin are other important markers in bone metabolism, involved in the RANK/RANKL/OPG and Wnt/β-catenin signaling pathways, respectively [14,15].

Conflicting results regarding bone formation and resorption markers have been observed in patients with PWS. Higher or unchanged bone turnover rates were found with low BMD and bone mineral content (BMC) values in obese adults with PWS without GH supplementation [16,17]. Other authors observed lower osteocalcin levels in association with a higher incidence of osteopenia and osteoporosis in overweight adults with PWS [18]. In youth with PWS, lower BMC and BMD values together with low bone alkaline phosphatase (BALP) levels but unchanged C-terminal telopeptide of type I collagen (CTX-I) levels were observed [19]. However, other studies on the RANK/RANKL/OPG and Wnt/ß-catenin signaling pathways in children with PWS have shown high bone turnover, which may be responsible for the altered BMD in these patients [5,20]. Currently, the treatment of children with PWS syndrome includes early treatment with GH, dietary recommendations, and behavioral intervention [2]. Despite improvements in the outcome of PWS patients, skeletal disorders remain a clinical problem. There is a lack of data on bone metabolism markers, including VKDPs, in children with PWS. Therefore, this study aimed to analyze biochemical bone markers, with particular emphasis on vitamin K-dependent proteins, in normal-weight children with PWS undergoing GH therapy and low-energy dietary intervention compared with healthy normal-weight children.

## 2. Results

Table 1 shows similar values of anthropometric parameters such as weight, height, BMI, BMI Z-score and fat mass in children with PWS and in healthy children. Lower values of total body less head-BMD (TBLH-BMD) (*p* = 0.018) and TBLH-BMD Z-score (*p* = 0.044) were found in children with PWS than in healthy children (Table 1).

**Figure 1:**
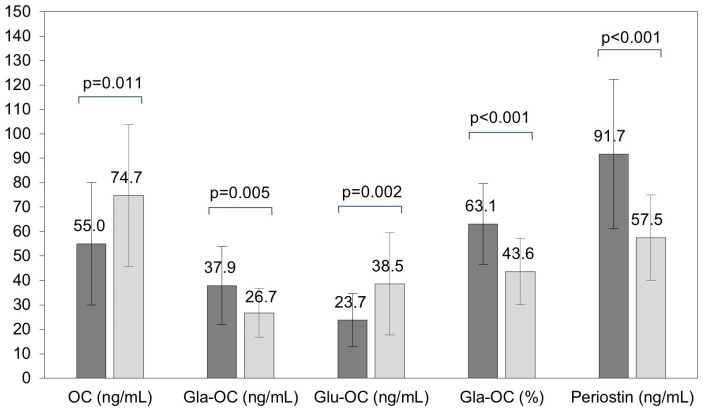
Concentrations of vitamin K-dependent proteins (VKDPs) in children with Prader–Willi syndrome (PWS) and in healthy children (dark grey—children with PWS, light grey—healthy children. Data are presented as mean values ± SD. OC—osteocalcin; Gla-OC—carboxylated osteocalcin; Glu-OC—undercarboxylated osteocalcin.

By analyzing the biochemical markers of bone turnover, we found similar values of BALP in both studied groups, but the levels of OC and the OC/CTX-I ratio were lower in children with PWS than in healthy children (*p* = 0.011, *p* = 0.006, respectively) (Table 2, Figure 1).

We found an altered profile of osteocalcin forms in patients compared with the controls. The Gla-OC concentrations were higher by about 30% (*p* = 0.005), but the Glu-OC concentrations were lower by about 30% (*p* = 0.002) in children with PWS than in healthy children. Hence, the percentage of Gla-OC was significantly higher in patients compared with the controls (*p* < 0.001). The periostin concentrations were 1.5-fold higher in these patients in comparison with healthy subjects (*p* < 0.001). Higher concentrations of OPN were also observed in patients with PWS (*p* = 0.032). No differences for CTX-I, sclerostin, and OPG concentrations were found between the studied groups.

The values of insulin-like growth factor-I (IGF-I) and the IGF-I/total-IGF-binding protein-3 (t-IGFBP-3) molar ratio were significantly higher in children with PWS than in the controls (*p* = 0.002; *p* < 0.001, respectively). Concentrations of 25-hydroxyvitamin D were higher in patients by about 50% (*p* < 0.001) compared with healthy children. Similar serum concentrations of parathyroid hormone (PTH), calcium, phosphorus, and magnesium were found in both studied groups.

Daily energy intake and the percentage of Estimated Energy Requirement (EER) were lower in children with PWS in comparison with healthy children by about 40% (*p* < 0.001) and 20% (*p* < 0.001), respectively (Table 3).

The percentage of energy from protein was higher (*p* < 0.001) but daily protein intake, including animal and plant proteins, was similar in both studied groups. The proportion of fat in the daily energy intake and daily fat intake were lower (*p* = 0.041; *p* = 0.001, respectively) in children with PWS than in the controls. The proportion of carbohydrates in the daily energy intake was similar in both studied groups, but daily carbohydrate intake was lower (*p* = 0.001) in patients in comparison with healthy children.

The diet of children with PWS contained a higher intake of vitamin D (*p* < 0.001), calcium (*p* < 0.001), and phosphorus (*p* = 0.028) than that of healthy children. The percentage of Adequate Intake (AI) for vitamin D and the percentage of Estimated Average Requirement (EAR) for calcium, phosphorus, and magnesium intake were also significantly higher in patients than in the controls (*p* < 0.001; *p* < 0.001; *p* = 0.039; *p* = 0.027, respectively). 

We used partial correlation to analyze associations between osteocalcin forms, periostin and other bone turnover markers in children with PWS and in healthy children after adjusting for age (Table 4).

We observed positive correlations between concentrations of osteocalcin and both forms of osteocalcin—Glu-OC and Gla-OC—in children with PWS (*p* = 0.002; *p* = 0.019, respectively). In the patient group, we obtained a positive correlation between concentrations of Gla-OC and percentage of Gla-OC (*p* = 0.001). We also found a positive correlation between the OC/CTX-I ratio and Glu-OC concentrations in this group (*p* = 0.035). Positive correlations between periostin and OC (*p* = 0.023), Gla-OC (*p* = 0.040), the OC/CTX-I ratio (*p* = 0.005), and OPG (*p* = 0.017) were also observed in patients with PWS. 

In healthy children, similar to the group with PWS, we also observed positive correlations between osteocalcin and both forms of osteocalcin—Glu-OC and Gla-OC (*p* < 0.001; *p* = 0.002, respectively). We did not find any associations between Gla-OC concentrations and the percentage of Gla-OC in the control group (*p* = 0.080). The OC/CTX-I ratio correlated positively with Glu-OC in this group (*p* = 0.001). However, opposite to children with PWS, we also found a negative correlation between this ratio and the percentage of Gla-OC (*p* = 0.012) in the control group. In healthy children, we also observed positive correlations between BALP activity and Glu-OC (*p* = 0.049) and negative correlations between BALP activity and the percentage of Gla-OC (*p* = 0.044). No significant correlations were found between periostin and other bone markers in this group. 

To adjust for age, the bivariate relationships between IGF-I and bone metabolism parameters, multivariate quantile regression models were estimated, including IGF-I and age as the independent variables and a single bone metabolism parameter as the dependent variable (Table 5).

The relationships between IGF-I and OC (*p* = 0.013), Gla-OC (*p* = 0.042) and the OC/CTX-I ratio (*p* = 0.017) were significant after adjustment for age in patients with PWS. In healthy children, the relationship between IGF-I and Gla-OC was only significant after adjustment (*p* = 0.008). No associations were obtained between the IGF-I/t-IGFBP-3 molar ratio and bone metabolism parameters in both studied groups.

## 3. Discussion

Current therapeutic strategies in PWS include early therapy with growth hormone (GH) in children to improve growth and body composition [23]. Baker et al. [3] demonstrated that the BMD of total body and the lumbar spine were stable in prepubertal children with PWS during GH therapy but decreased in adolescents. However, reduced lumbar spine BMD in children with PWS receiving GH was found by Brunetti et al. [5]. In addition, Oto et al. [4] observed that total body BMD of pediatric patients with PWS decreased gradually despite GH treatment. Our results also show lower TBLH BMD and TBLHBMD Z-score values in prepubertal normal-weight children with PWS during GH therapy and dietary intervention than in healthy children. In addition, we observed changes in the profile of biochemical bone turnover markers in the studied patients in comparison with healthy subjects. The lower OC concentrations and OC/CTX-I ratio suggest that bone turnover disorders may result from impaired bone formation in children with PWS. We also found higher concentrations of osteopontin in these patients, which is produced together with osteocalcin during bone formation in the late phase of mineralization. OPN expression is strongly dependent on PTH and proinflammatory cytokines [24]. Higher levels of this protein have been observed in osteoporosis, osteoarthritis, obesity, and diabetes [25,26,27]. Despite unchanged PTH levels and normal body weight in our children with PWS, we observed higher OPN levels in these patients, which may predispose them to increased bone resorption. Furthermore, in our previous study [28] we found associations between proinflammatory adipokines and the prooxidant state in non-obese children with PWS, which may also influence OPN levels in these patients [29].

Studies concerning bone markers in children and adolescents with PWS are rather scarce. Rubin et al. [19] obtained unchanged BALP and CTX-I values in youths with PWS during pharmacotherapy and exercises compared with youths without PWS. However, Brunetti et al. [5] found high osteocalcin levels and low 25(OH)D levels associated with the reduced BMD in children with PWS. These authors also observed the occurrence of higher bone turnover and increase in the RANKL/OPG ratio in the studied patients. Brunetti et al. [20] suggest that vitamin D supplementation could improve BMD in children with PWS. In our study, children with PWS were supplemented with vitamin D, and serum concentrations of 25(OH)D were significantly higher in patients than in healthy children. In addition, children with PWS had not only a higher intake of vitamin D but also a higher percentage of EAR for calcium, phosphorus, and magnesium intake than the control group. However, we found lower values of total osteocalcin as well as alterations in the profile of OC forms with higher Gla-OC concentrations but lower Glu-OC concentrations in children with PWS compared with healthy subjects. Gla-OC is a marker of mature osteoblasts and may interact with hydroxyapatite crystals, modulating its growth, but elevated serum Glu-OC levels are associated with lower BMD, increased osteoporosis, fracture risk, and vitamin K deficiency [30,31]. The exact role of these proteins in bone metabolism is not entirely clear. Ziemińska et al. [32] observed an unfavorable role of Gla-OC in the mineralization of bones in chronic kidney disease conditions. According to other authors, Gla-OC may act as an inhibitor of bone mineralization [33,34]. In our study, the higher percentage of Gla-OC and low Glu-OC concentrations may indicate higher levels of serum vitamin K and more intensive carboxylation processes of vitamin K-dependent proteins in children with PWS. Moreover, these alterations in the profile of OC forms and lower OC concentrations were obtained together with lower bone densities in these children in comparison with healthy subjects. It seems that the role of OC and OC forms in bone formation disorders in children with PWS cannot be ruled out.

Furthermore, decreased Glu-OC concentrations may be an early symptom of metabolic syndrome and insulin resistance in humans and mice [35,36]. Takaya et al. [37] observed decreased Glu-OC concentrations in non-obese children with type 2 diabetes mellitus and suggested a role of this protein in the pathophysiology of diabetes. Our previous results showed similar values of glucose and proinsulin in non-obese children with PWS during dietary treatment and in healthy subjects [22]. However, the low Glu-OC concentrations observed in these patients may be one of the factors predisposing them to the development of diabetes in the future. Particularly, when an appropriate low-calorie diet is not followed, individuals with PWS display hyperphagia, develop obesity, and a number of complications such as dyslipidemia, metabolic syndrome, glucose metabolism disorders, and type 2 diabetes [38].

Periostin, a protein also dependent on vitamin K, is an important regulator of collagen cross-linking, and loss of periostin changes the bone microarchitecture and reduces bone strength and turnover [39]. Higher serum periostin concentration is associated with higher fracture risk in postmenopausal women [8,40] and may be used as a marker for spinal degenerative diseases [41]. Other authors also observed increased periostin levels in fractures during the early healing phase, suggesting the participation of this protein in bone repair processes [42]. According to Chapurlat et al. [43], higher concentrations of periostin may reflect adaptation of periosteum cell metabolic activity in response to different disorders to maintain a steady state of skeletal system quality. In our study, we observed higher concentrations of periostin in children with PWS than in the healthy controls and significant positive relations with osteocalcin and Gla-OC, which may be the result of increased protein carboxylation processes dependent on vitamin K. Additionally, periostin stimulates the Wnt signaling pathway, inhibiting the expression of sclerostin to mediate the anabolic response of the bone [7]. We did not find differences in sclerostin and OPG concentrations between children with PWS and the controls. OPG has been shown to reduce osteoclast number, which affects the rate of bone resorption [44]. Previous studies have shown that PTH can affect OPG expression [45], but in our study unchanged PTH and OPG values in children with PWS were found. Furthermore, we observed a significant correlation between OPG and periostin in our patients. Some authors showed positive associations between these markers during tooth eruption in mice [46] as well as during cell adhesion to implantable materials and osteoblastic differentiation on implant surfaces [47]. It seems that periostin together with OPG may play a compensatory role in response to bone metabolic disorders in patients with PWS.

In our study, we observed higher concentrations of IGF-I and the IGF-I/t-IGFBP-3 ratio in children with PWS during GH treatment in comparison with healthy subjects. Similar results were obtained by Baker et al. [48] and Gaddas et al. [49] in GH-treated PWS children who had elevated serum concentrations of IGF-I and IGFBP-3 in the upper normal range. Additionally, an increase in IGF-I concentrations and a relatively smaller increase in IGFBP-3 concentrations were observed in these patients, which may result in a greater amount of free, biologically available IGF-I in the circulation [49]. Animal models and human studies showed that IGF-I deficiency as well as IGF-I excess may cause disorders in bone metabolism, resulting in alterations in bone mass and skeletal development [50,51]. We observed positive relations between IGF-I and Gla-OC in both studied groups and additionally between IGF-I and OC as well as the OC/CTX-I ratio in children with PWS. However, the role of increased IGF-I concentrations in bone mineral density and skeletal development in patients with PWS is still unclear. Therefore, the relation between the GH/IGF-I axis and bone metabolism parameters, especially vitamin K-dependent proteins, requires further research.

The present study had several limitations. First, we analyzed bone turnover markers and bone density in a small group of normal-weight children with PWS, but this syndrome is a rare genetic disease. However, all children in the study group were treated with GH and a low-energy diet and were within the normal range in terms of BMI. Secondly, we presented a cross-sectional study without a prospective longitudinal analysis, which is needed to examine the relationship between bone metabolism parameters and clinical outcomes in children with PWS. However, this is the first study investigating bone turnover markers and vitamin K-dependent proteins in children with PWS. Thirdly, we did not directly measure blood concentrations of vitamin K and its metabolites in these patients. According to many authors, the percentage of Gla-OC or the Gla-OC/Glu-OC ratio may also be useful in determining vitamin K status [10]. Finally, we know that myokines—i.e., factors released by muscle tissue—have a significant impact on bone metabolism. These factors can be released by skeletal muscle into the circulatory system in response to mechanical stress and/or metabolic changes. Fibroblast growth factor-2 (FGF-2) and irisin stimulate bone growth by increasing bone formation. In contrast, bone resorption can be modified by the action of myostatin and fibroblast growth factor-21 (FGF-21). Therefore, our further research will focus on the influence of selected myokines on the processes of bone formation and resorption in children with PWS.

## 4. Materials and Methods

### 4.1. Patients

**Figure 2:**
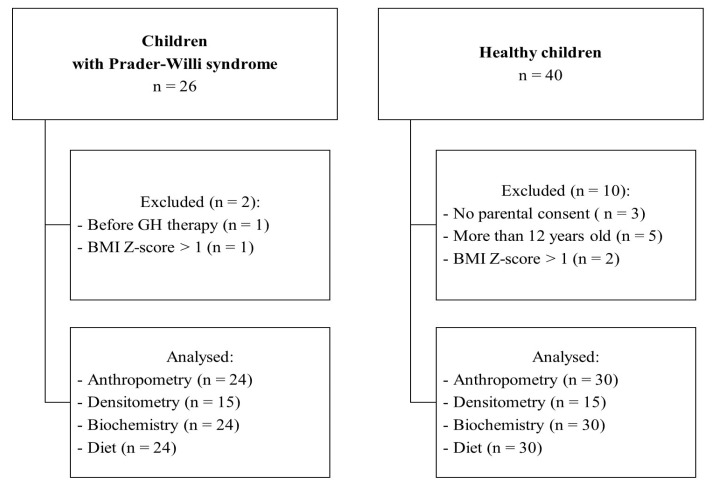
Flowchart of the study. BMI—body mass index; GH—growth hormone.

Twenty four normal-weight children with PWS aged 2–12 years and 30 healthy children of the same age were recruited between 2022 and 2024. As described previously, patients with PWS were included if they had a genetically confirmed diagnosis of PWS, treatment with GH for at least one year as well as a low-energy diet at the time of inclusion [22]. Patients with PWS were excluded if they had a chronic secondary illness such as diabetes mellitus, liver, or kidney diseases. The healthy children were included in the control group with a BMI Z-score <−1 + 1> and within the same age range as the group of patients (Figure 2).

The dietary guidelines, recommending a low-energy diet based on a balanced distribution of carbohydrates, proteins, and lipids for children with PWS, were described in a previous study [22]. The recommended reduction in daily energy intake for these patients was 20–40%. Average daily food rations and their nutritional value were calculated using nutritional analysis software Dieta 5^®^ (extended version Dieta 5.0, National Food and Nutrition Institute, Warsaw, Poland) [52]. The age- and sex-specific percentage of EER for total energy intake, EAR for calcium, phosphorus and magnesium, and AI for vitamin D were calculated for each patient with PWS and each healthy child. The children in the present study only received standard supplementation with vitamin D.

Physical examinations, including body height and weight measurements, were performed in both groups. The BMI of each individual was converted to a standard BMI Z-score for the child’s age and sex using Polish reference tables [53]. Body composition, total BMC, and BMD were measured by dual-energy X-ray absorptiometry (DXA) using Lunar Prodigy (General Electric Healthcare, Madison, WI) with pediatric software 9.30.044. 

Written informed consent was obtained from the parents of all the examined children. The study was performed in accordance with the Helsinki Declaration for Human Research, and the study protocol was approved (protocol code: 17/2022; date of approval: 5 May 2022) by the Ethics Committee of the Institute of Mother and Child in Warsaw, Poland.

### 4.2. Biochemical Methods

Venous blood was taken after an overnight fast in the morning (between 8:00 AM and 10.00 AM) for biochemical analyses. Serum samples were collected after centrifugation (1000× *g* for 10 min at 4 °C) and stored frozen (−70 °C) until analysis (no longer than six months). Biochemical parameters were determined by immunoenzymatic methods. BALP activity was estimated using the BAP EIA kit from Quidel (Athens, OH, USA) with a within-assay variability of less than 5.8% and a between-assay variability of less than 7.6%. OC and CTX-I concentrations were measured with the N-MID Osteocalcin ELISA kit and serum CrossLaps ELISA kit (IDS, Bolton, UK), respectively. The intra-and inter-assay coefficients of variation were less than 2.2% and 5.1% for OC and 3.0% and 10.9% for CTX-I, respectively. The detection limit was 0.7 U/L for BALP, 0.5 ng/mL for OC, and 0.02 ng/mL for CTX-I.

Levels of Gla-OC and Glu-OC in serum were measured using kits from Takara Bio Inc. (Shiga, Japan), which had intra- and inter-assay CVs of less than 4.8% and 2.4% for Gla-OC, and 6.7% and 9.9% for Glu-OC, respectively. The detection limit was 0.25 ng/mL for both forms of OC.

The sclerostin concentrations were measured using a Sclerostin HS kit from Teco Medical Group (Sissach, Switzerland), which had inter- and intra-assay CVs of less than 4.8% and 8.2%, respectively. Serum OPG, OPN and periostin levels were determined using the ELISA kit from DRG (Marburg, Switzerland), FineBiotech (Wuhan, China), and AdipoGen Life Science (Liestal, Switzerland), respectively. Intra- and inter-assay variations were less than 4.9% and 9.0% for OPG, 8.0% and 10.0% for OPN, and 8.6% and 9.9% for periostin, respectively. The detection limit was 0.006 ng/mL for sclerostin, 0.03 pmol/L for OPG, 0.094 ng/mL for OPN, and 15 pg/mL for periostin.

Commercially available ELISA kits from Mediagnost (Reutlingen, Germany) were used to determine concentrations of IGF-I and t-IGFBP-3. The intra- and inter-assay coefficients of variation were less than 6.7% and 6.6% for IGF-I, and 2.2% and 7.4% for t-IGFBP-3, respectively. We calculated the IGF-I/IGFBP-3 molar ratio as [IGF-I (ng/mL) × 0.130]/[IGFBP-3 (ng/mL) × 0.036]. The detection limit was 0.091 ng/mL for IGF-I and 0.15 ng/mL for t-IGFBP-3.

Serum levels of 25-hydroxyvitamin D and PTH were determined by the electrochemiluminescence method (ECLIA) with kits from DiaSorin Inc. (Stillwater, MN, USA). The calcium, phosphorus, and magnesium concentrations were measured using standard methods (Roche Diagnostics, Basel, Switzerland).

### 4.3. Statistical Analyses

The Kolmogorov-Smirnov test was used to evaluate distribution for normality. The obtained results are presented as means ± standard deviation (SD) for normally distributed data or medians and interquartile range (25th–75th percentiles) for non-normally distributed variables. Differences in anthropometric characteristics, dietary intake and bone turnover markers between children with PWS and healthy children were assessed using Student’s *t*-test or the Mann-Whitney test based on the above assumptions. Using Student’s *t*-test, equity of variances was taken into account. For group *n* = 15, the exact Mann–Whitney test was applied.

Bivariate relationships were assessed using Pearson’s correlation for normally distributed dependent variables or quantile regression for non-normally distributed variables. To adjust for age, partial correlations or quantile regression with age as one of the independent variables were estimated. A *p*-value of < 0.05 was considered to be statistically significant. Statistical analysis was performed using IBM SPSS v.25.0 software (SPSS Inc., Chicago, IL, USA) and Stata Rel.18 (StataCorp LLC, College Station, TX, USA).

## 5. Conclusions

In this study, bone turnover disorders in normal-weight children with PWS undergoing GH treatment and dietary intervention may result from impaired bone formation due to the lower concentrations of OC and the OC/CTX-I ratio. Furthermore, we observed associations between Gla-OC and IGF-I concentrations in children with PWS. The altered profile of OC forms with a higher percentage of Gla-OC, along with elevated periostin concentrations, may indicate more intensive carboxylation processes of vitamin K-dependent proteins in these patients.

## Figures and Tables

**Table 1:** Anthropometric characteristics in children with Prader–Willi syndrome (PWS) and in healthy children.

	Children with PWS *n* = 24	Healthy Children *n* = 30	*p*-Value
Age (year)	6.1 ± 3.1	6.5 ± 3.0	0.586
Girls/Boys	12/12	15/15	−
Height (cm)	113.1 ± 22.9	117.2 ± 18.7	0.470
Weight (kg)	20.3 ± 9.2	22.2 ± 8.3	0.425
BMI (kg/m^2^)	15.2 ± 1.6	15.7 ± 1.6	0.234
BMI Z-score	−0.54 ± 0.71	−0.26 ± 0.63	0.128
	*n* = 15	*n* = 15	
Fat mass (%)	20.6 ± 4.2	19.1 ± 4.8	0.992
Fat mass (kg)	5.12 ± 2.0	3.86 ± 1.3	0.061
TBLH-BMC (kg)	0.57 ± 0.17	0.47 ± 0.15	0.089
TBLH-BMD (g/cm^2^)	0.57 ± 0.07	0.64 ± 0.06	0.018
TBLH-BMD Z-score	−0.95 ± 0.54	−0.48 ± 0.69	0.044

Data are presented as mean values ± SD. BMI—body mass index; TBLH—total body less head; BMC—bone mineral content; BMD—bone mineral density.

**Table 2:** Biochemical measurements in children with PWS and in healthy children.

	Children with PWS *n* = 24	Healthy Children *n* = 30	*p*-Value
BALP (U/L)	126.3 ± 34.9	126.3 ± 39.4	0.996
Osteopontin (ng/mL)	59.3 (48.9–85.8)	50.6 (35.3–67.5)	0.032
OC/CTX-I	0.35 ± 0.15	0.46 ± 0.15	0.006
CTX-I (ng/mL)	1.68 ± 0.52	1.64 ± 0.45	0.813
OPG (pmol/L)	4.28 ± 0.84	4.56 ± 1.28	0.361
Sclerostin (ng/mL)	0.45 ± 0.26	0.42 ± 0.18	0.646
IGF-I (ng/mL)	299.6 (136.8–333.0)	134.4 (108.2–194.5)	0.002
IGF-I/t-IGFBP-3 molar ratio	0.24 ± 0.08	0.16 ± 0.06	<0.001
PTH (pg/mL)	18.4 ± 9.2	20.5 ± 9.0	0.414
25-hydroxyvitamin D (ng/mL)	35.9 ± 9.0	23.9 ± 8.6	<0.001
Calcium (mmol/L)	2.59 ± 0.09	2.59 ± 0.07	0.970
Phosphorus (mmol/L)	1.67 ± 0.15	1.72 ± 0.10	0.155
Magnesium (mmol/L)	0.89 ± 0.05	0.90 ± 0.02	0.112

Data are presented as mean values ± SD or median values (25th–75th percentiles). BALP—bone alkaline phosphatase; CTX-I—C-terminal telopeptide of type I collagen; OPG—osteoprotegerin; IGF-I—insulin-like growth factor-I; t-IGFBP-3—total-IGF-binding protein-3; PTH—parathyroid hormone.

**Table 3:** Daily energy and nutrient intake of the examined children with PWS and the control group.

	Children with PWS *n* = 24	Healthy Children *n* = 30	*p*-Value
Energy (kcal/day)	1033 ± 322	1453 ± 396	<0.001
Energy (% of EER)	70.5 ± 14.5	93.2 ± 19.9	<0.001
Proteins (% of energy)	17.5 ± 4.4	13.4 ±2.3	<0.001
Carbohydrates (% of energy)	51.3 ± 7.1	53.7 ± 5.5	0.164
Fat (% of energy)	29.7 ± 5.5	32.8 ± 5.2	0.041
Protein (g/day)	45.5 ± 18.7	46.7 ± 17.8	0.817
Animal protein (g/day)	31.4 ± 15.3	30.4 ± 11.3	0.787
Plant protein (g/day)	13.4 ± 5.9	16.2 ± 6.9	0.122
Carbohydrates (g/day)	144.7 ± 45.9	197.7 ± 65.4	0.001
Fat (g/day)	35.0 ± 14.5	51.3 ± 19.6	0.001
Vitamin D (µg/day)	8.08 (3.09–15.31)	1.90 (1.32–2.47)	<0.001
Vitamin D (% of AI)	54 (19–102)	13 (9–17)	<0.001
Calcium (mg/day)	795.5 ± 261.3	508.7 ± 272.2	<0.001
Calcium (% of EAR)	113 ± 41	63 ± 32	<0.001
Phosphorus (mg/day)	1004.3 ± 313.2	805.8 ± 310.5	0.028
Phosphorus (% of EAR)	209 ± 62	168 ± 75	0.039
Magnesium (mg/day)	204.9 ± 76.3	182.5 ± 76.3	0.299
Magnesium (% of EAR)	202 ± 62	160 ± 67	0.027

Data are presented as mean values ± SD or median values (25th–75th percentiles). EER—Estimated Energy Requirement; EAR—Estimated Average Requirement; AI—Adequate Intake. Recommended daily energy and nutrient intakes were calculated according to Jarosz [21] and described by Gajewska et al. [22].

**Table 4:** Relations between osteocalcin forms, periostin and bone turnover markers in children with PWS and in healthy children.

		**Children with PWS**			
		Glu-OC	Gla-OC	Gla-OC%	Periostin
OC	r (*p*)	0.580 (0.003)	0.512 (0.010)	−0.195 (0.360)	0.446 (0.029)
	partial r (*p*)	0.613 (0.002)	0.485 (0.019)	−0.225 (0.303)	0.473 (0.023)
Gla-OC	r (*p*)	−0.006 (0.976)	X	0.640 (0.001)	0.419 (0.042)
	partial r (*p*)	−0.003 (0.988)	X	0.642 (0.001)	0.434 (0.040)
OC/CTX-I	r (*p*)	0.433 (0.035)	0.412 (0.045)	−0.021 (0.921)	0.553 (0.005)
	partial r (*p*)	0.441 (0.035)	0.394 (0.063)	−0.031 (0.888)	0.562 (0.005)
BALP	r (*p*)	−0.105 (0.627)	0.217 (0.309)	0.130 (0.545)	−0.118 (0.584)
	partial r (*p*)	−0.104 (0.637)	0.214 (0.327)	0.128 (0.560)	−0.117 (0.594)
OPG	r (*p*)	0.172 (0.421)	−0.181 (0.398)	−0.121 (0.524)	0.480 (0.018)
	partial r (*p*)	0.174 (0.427)	−0.136 (0.536)	−0.109 (0.622)	0.494 (0.017)
Sclerostin	r (*p*)	0.366 (0.079)	0.325 (0.121)	−0.061 (0.775)	0.083 (0.700)
	partial r (*p*)	0.274 (0.206)	0.274 (0.206)	−0.094 (0.670)	0.098 (0.657)
		**Healthy Children**			
		Glu-OC	Gla-OC	Gla-OC%	Periostin
OC	r (*p*)	0.846 (<0.001)	0.216 (0.253)	−0.590 (0.001)	0.060 (0.752)
	partial r (*p*)	0.752 (<0.001)	0.551 (0.002)	−0.333 (0.077)	0.154 (0.443)
Gla-OC	r (*p*)	−0.0003 (0.999)	X	0.451 (0.012)	0.260 (0.165)
	partial r (*p*)	0.287 (0.131)	X	0.331 (0.080)	0.242 (0.206)
OC/CTX-I	r (*p*)	0.677 (<0.001)	−0.012 (0.948)	−0.592 (0.001)	−0.064 (0.736)
	partial r (*p*)	0.579 (0.001)	0.152 (0.433)	−0.458 (0.012)	−0.024 (0.902)
BALP	r (*p*)	0.311 (0.094)	−0.013 (0.945)	−0.318 (0.087)	0.131 (0.492)
	partial r (*p*)	0.368 (0.049)	0.001 (0.995)	−0.377 (0.044)	0.136 (0.483)
OPG	r (*p*)	−0.218 (0.247)	0.095 (0.618)	0.253 (0.178)	−0.061 (0.749)
	partial r (*p*)	−0.129 (0.504)	0.035 (0.858)	0.174 (0.366)	−0.082 (0.674)
Sclerostin	r (*p*)	−0.116 (0.542)	−0.008 (0.996)	0.145 (0.445)	−0.062 (0.743)
	partial r (*p*)	−0.308 (0.104)	0.057 (0.767)	0.349 (0.064)	−0.045 (0.817)

BALP—bone alkaline phosphatase; OC—osteocalcin; Glu-OC—undercarboxylated osteocalcin; Gla-OC—carboxylated osteocalcin; OPG—osteoprotegerin; CTX-I—C-terminal telopeptide of type I collagen.

**Table 5:** Crude and age-adjusted associations between insulin-like growth factor-I (IGF-I) and bone markers in children with PWS and in healthy children (quantile regression).

	Children with PWS					
	Crude			Age-Adjusted		
	Beta	95%CI	*p*-Value	Beta	95% CI	*p*-Value
OC	0.08	−0.20–0.18	0.111	0.16	0.04–0.28	0.013
Gla-OC	0.08	−0.003–0.16	0.059	0.12	0.004–0.23	0.042
Glu-OC	−0.02	−0.07–0.03	0.469	0.04	−0.03–0.10	0.252
Gla%	−0.002	−0.08–0.08	0.964	0.03	−0.10–0.16	0.690
OC/CTX-I	0.001	−0.00002–0.001	0.058	0.0009	0.0002–0.002	0.017
Periostin	0.10	−0.03–0.23	0.127	0.15	−0.04–0.34	0.122
**Healthy Children**						
OC	0.19	0.06–0.32	0.006	0.11	−0.07–0.29	0.215
Gla−OC	0.01	−0.04–0.06	0.669	0.08	0.02–0.14	0.008
Glu-OC	0.12	0.01–0.22	0.032	0.10	−0.03–0.23	0.134
Gla%	−0.05	−0.11–0.02	0.145	0.02	−0.05–0.09	0.544
OC/CTX-I	0.0003	−0.0001–0.0009	0.124	0.0002	−0.0009–0.001	0.723
Periostin	0.02	−0.07–0.11	0.686	0.02	−0.12–0.16	0.733

OC—osteocalcin; Glu-OC—undercarboxylated osteocalcin; Gla-OC—carboxylated osteocalcin; CTX-I—C-terminal telopeptide of type I collagen.

## Data Availability

The data presented in this study are available upon reasonable request to the corresponding author.
